# Comparison between Two Types of Dental Unit Waterlines: How Evaluation of Microbiological Contamination Can Support Risk Containment

**DOI:** 10.3390/ijerph16030328

**Published:** 2019-01-24

**Authors:** Jessica Lizzadro, Marta Mazzotta, Luna Girolamini, Ada Dormi, Tiziana Pellati, Sandra Cristino

**Affiliations:** 1 Department of Biological, Geological, and Environmental Sciences, BiGeA, University of Bologna, via San Giacomo 12, 40126 Bologna, Italy; jessica.lizzadro2@unibo.it (J.L.); marta.mazzotta2@unibo.it (M.M.); luna.girolamini2@unibo.it (L.G.); 2 Department of Medical and Surgical Science, DIMEC, University of Bologna, via San Giacomo 12, 40126 Bologna, Italy; ada.dormi@unibo.it; 3 GVM Care & Research, via Emaldi 10, 48124 Lugo di Ravenna, Italy; tpellati@gvm-engineering.it

**Keywords:** water quality, dental unit waterline (DUWL), heterotrophic plate counts (HPCs), *Pseudomonas aeruginosa*, *Legionella*, risk containment

## Abstract

Infection risk management in a dental unit waterline (DUWL) involves healthcare personnel and patients and is related to routine exposure to water and aerosols that may contain bacterial species. To improve water safety plans, maintenance, and sanitation procedures, analyses of heterotrophic plate counts (HPCs) at 36 °C, and two other microorganisms frequently associated with biofilms, *Pseudomonas aeruginosa* and *Legionella* spp., were performed in order to evaluate differences in microbiological contamination between two types of DUWLs: Type A, provided by a water tank, and Type B, directly connected to municipal water. The data showed that the water supply and water safety plan differentially influenced microbiological contamination: Type A DUWLs were more contaminated than Type B DUWLs for all microbiological parameters tested, with significant changes in the percentage of positive samples and contamination levels that were beyond the limits of standard guidelines. The results obtained show how the storage tank, the absence of anti-retraction valves, and the disinfection procedures performed are the main critical points of Type A DUWLs, which confirms that dental unit management (maintenance/sanitization) is often missed or not correctly applied by stakeholders, with an underestimation of the real risk of infection for patients and operators.

## 1. Introduction

A dental unit waterline (DUWL) is a complex system that delivers water to different points: water bottle tanks, glasses for patients, handpieces for high-speed drills, ultrasonic scalers, and air and water syringes. Generally, DUWLs are equipped with a dual water supply system that permits the system to be supplied with only municipal water or sterile water or with both types. The use of different types of water is regulated by a bypass button on the control panel and is associated with the water volume requirement or is chosen by medical staff. The presence of bacteria in DUWLs, water, and aerosols produced by dental procedures was reported for the first time by Blake in 1963 [1,2].

DUWLs represent a potential vehicle of infection and pose a risk of infection for not only dental patients (especially immunocompromised patients) but also healthcare personnel, who are routinely exposed to the same risk.

Dental infections can be triggered by direct exposure to human pathogens from the oral cavities of patients. These pathogens are spread after oral fluids are aspirated and/or are transferred from contaminated handpieces or instruments [3]. Moreover, the use of water is required for most dental procedures. Therefore, the water should be of good quality based on the recommended quality levels for human consumption [4]. Water quality is also an indispensable requirement for minimizing the risk of exposure and avoiding infections related to healthcare procedures [5]. The main water-related infections in DUWLs are of bacterial origin because bacteria represent the dominant part of the microflora, even though many studies have shown the presence of fungi and protozoa [6,7,8]. The most common contaminants of DUWLs are Gram-negative aerobic environmental species that are not pathogenic in healthy individuals but may be harmful to the respiratory systems of immunocompromised patients (e.g., *Legionella*, *Pseudomonas aeruginosa,* and nontuberculosis Mycobacterium) [9,10,11].

Uzel et al. [12] demonstrated that, among the microbiological contaminants of DUWLs, Pseudomonadaceae species, including *Burkholderia cepacia*, *Chryseomonas luteola*, *Pseudomonas fluorescens*, *Ralstonia pickettii,* and *Sphingomonas paucimobilis*, are the most highly represented among isolated and identified microorganisms.

Microbial proliferation within DUWLs is inevitable because a DUWL consists of a complex and extended system of narrow-bore, small-diameter tubes (approximately 2 to 3 mm internal diameter) in plastic material [13].

In addition, the laminar flow of water passing through a DUWL is maximal at the center of the lumen and less at the periphery, which favors the deposition and adhesion of microorganisms to the surface of the tube and, thus, promotes biofilm formation [3,14,15]. Biofilms represent a complex community of bacteria within an extracellular polysaccharide matrix [10]. The presence of biofilms in DUWLs is related to several factors, such as water stagnation, which occurs as a result of inactivity, mostly in the evening, during the night, or during weekends or holidays, anti-retraction valves failure, the presence of water heaters (maintaining temperatures over 20 °C), and variations in the type of water supply (tap water, distilled water, or sterile water) [9,16]. Once the biofilm is formed, it serves as a continuous reservoir of bacteria in a DUWL [17].

Numerous approaches have been introduced to decrease the presence of DUWL biofilms, including both nonchemical and chemical methods. In terms of nonchemical strategies (including flushing, drying, and applying an antimicrobial filter), deionized, distilled, or even sterile water does not appear to affect existing biofilms [17,18].

Flushing out the water from handpieces (high-speed drills, ultrasonic scalers, and air and water syringes) is useful for eliminating the stagnant liquid in the pipes after an inactive period (for example, at the beginning of the work day) because it generates a pressure suitable for removing bacteria that weakly adhere to the biofilm.

The application of chemical agents has been demonstrated to be more effective than nonchemical methods. Moreover, the synergistic effects of the two types of methods have been evaluated by different studies and have shown better performance than single methods [19,20,21].

The scientific literature reports the use of different disinfection methods, particularly those based on chemical approaches, such as treatment with sodium hypochlorite and hydrogen peroxide with or without silver ions and peracetic acid. These methods have the following disadvantages: intermittent treatment has a transient action that facilitates recolonization, the continuous use of disinfectants promotes the risk of exposure to biocidal agents among patients and staff, and long-term treatment can increase the risk of resistance development by bacterial strains [3,22].

Therefore, disinfection treatments are not effective if they are not correctly performed to ensure the best dosage and contact time.

DUWL manufacturers’ suggestions are based on limited information regarding this issue and include guaranteeing the recirculation of water in a water system, water flushing, or, sometimes, the use of sterile tanks or anti-retraction valves to avoid retro-contamination by handpieces. The manufacturers also suggest the application of certain disinfection procedures, such as effervescent tablets and continuous disinfectant circulation, associated with the use of sterilization protocols applied to handpieces between each patient [23,24,25].

To date, there is no unique international regulation that establishes procedures for cleaning, control, and disinfection of DUWLs, and there are no limits for microbial concentrations. The Center for Disease Control (CDC) recommends monitoring water quality by using disinfection products and devices that guarantee microbiological quality standards, i.e., ≤500 colony-forming units (cfu)/mL of heterotrophic water bacteria, the consultation of instruction manuals provided by the manufacturers for the maintenance of DUWLs, the use of sterile saline or sterile water for surgical procedures, and the performance of a flushing procedure between patients at the start of each working session. Therefore, DUWLs should be flushed, drained, and left disconnected during any temporary closure [26].

Starting with this knowledge and following the adoption of a water safety plan for drinking water in dental units of five hospitals located in northern Italy, we have undertaken environmental monitoring of water quality indicators and pathogenic bacteria such as heterotrophic plate counts (HPCs) at 36 °C, *Pseudomonas aeruginosa* and *Legionella*. The monitoring was performed by comparing two different types of DUWLs: dental units frequently supplied by a storage tank (called “Type A”) and dental units always supplied by tap water (called “Type B”). The aim of the study was to establish the source of DUWL contaminants and identify the critical points related to the dental environment. The data obtained were also compared with different guidelines to assess their compliance and implement a water safety plan related to the application of disinfection and maintenance protocols.

## 2. Materials and Methods

### 2.1. DUWL Characteristics

Fourteen DUWLs were selected from 5 hospitals provided with a water safety plan to assess the microbiological characteristics of the water supply.

The DUWLs were divided into two types: Type A (*n* = 10), with a closed water supply system fed only by a bottle storage tank (1- or 2-L volume) filled with sterile or distilled water, and Type B (*n* = 4), with an open water supply system connected to municipal water. The characteristics of the two types of DUWLs and the maintenance procedure adopted are presented in Table 1.

#### 2.1.1. Type A

Type A DUWLs are equipped with a dual water supply system including the municipal water network and an independent bottle tank that feeds the handpieces and the patient’s glass. The tank has a variable capacity that depends on the model of the dental unit (1 L or 2 L) and is filled with either sterile or distilled water, according to the indication provided by the manufacturers. The use of the bottle tank is regulated by a bypass button on the control panel (Table 1), and this button permits bypassing the bottle tank and supplying the DUWL directly with tap water. This type of dental unit is usually preferred when invasive dental surgery is performed. Depending on the capacity of the bottle tanks, manufacturers recommend sanitization procedures for the entire circuit, based on the use of cleaning products approved by the guidelines, which must be performed periodically, typically weekly, at the end of the working day and after long periods of inactivity (weekends or holidays). Regarding disinfection products, a solution containing sodium percarbonate (6.96%), dimethyl benzyl ammonium chloride (0.85%), dimethyl ethylbenzyl ammonium chloride (0.85%), silver nitrate (0.14%), and other ingredients (91.2%) is used in the form of effervescent tablets dissolved in 1 or 2 L of bottle tank water. The disinfection activity remains stable for up to two weeks, according to the manufacturer’s manual [27]. The companies also recommend microbiological analysis to test the water quality after each disinfection treatment. As suggested by the manufacturer’s manual, the water quality of DUWLs can be maintained by flushing all the pipes that supply the instruments before starting work and at the end of the working day.

#### 2.1.2. Type B

Type B DUWLs are connected directly to the main municipal water system through a pipeline network and do not require the use of an independent tank. These DUWLs are equipped with an automated sanitation system in which a concentrated disinfectant (supplied by the manufacturer) is placed inside a container and passes through a second container, where it is diluted and mixed with distilled water. The mixing operations are controlled by an electronic system and lead to the delivery of sanitized water that flows into the instrument tubes. This type of DUWL is applied for each type of dental procedure. Sanitizing products are composed of hydrogen peroxide and silver salt (3% *v*/*v*). The company recommends the use of only authorized products covered by patents. The washing of DUWLs is performed at the end of each operation by using sterile water to complete the cleaning and discarding of the disinfectant residues. The system is flushed at the beginning and at the end of each working day as well as after a long-term closed circuit.

### 2.2. Water Sample Collection

Microbiological parameters were analyzed in both types of DUWLs by collecting samples from the bottle tanks and handpieces (for Type A) and from the glasses of patients and handpieces (for Type B). Sampling was also performed in some outlets close to the dental chair to test the quality of the municipal water. All samples were taken at the beginning of the working day.

In the case of Type A, the total volume of the bottle tank water (1 or 2 L) was transferred to sterile polytetrafluoroethylene (PTFE) bottles containing a sodium thiosulfate solution (10% *v*/*v*) to neutralize any residual disinfectant, while the handpieces samples from all supplied instruments (high-speed drills, ultrasonic scalers, air-water syringes), with variable volumes from 50 to 100 mL, were collected and mixed together in PTFE bottles.

For Type B, water sampling (1 or 2 L) from the glasses of patients was performed directly in a PTFE bottle, while sampling from handpieces was performed as previously described for Type A.

More specifically, during the sampling from the glasses of patients and handpieces, the water was run for 3 to 5 min to discard stagnant water and obtain representative samples of water flowing through the whole pipeline network, as suggested by Hwang et al. [28]. A total of 100 water samples were taken and analyzed for HPCs at 36 °C, *P. aeruginosa* and *Legionella* spp., including 84 samples from 10 Type A DUWLs and 16 samples from 4 Type B DUWLs. Due to the diverse clinical operations, the DUWLs were not sampled on the same day, but sampling followed the risk assessment plan for each DUWL. Some units, particularly Type A DUWLs, were sampled more than others when noncompliant results were obtained. This procedure was also performed to control compliance with the disinfection procedures undertaken.

### 2.3. Microbiological Analysis

HPCs at 36 °C were isolated using the standard plate method on tryptic glucose yeast agar (Plate Count Agar -PCA-, Biolife, Milan, Italy), according to UNI EN ISO 6222:2001 [29]. Each sample was processed in duplicate. Bacterial concentrations were determined by colony counts and expressed as cfu/mL.

The samples were considered noncompliant (positive) when the bacterial concentration exceeded the limits set by the CDC (i.e., ≤500 cfu/mL) [26] or the Italian drinking water regulation, Decreto legislativo 31/2001 (i.e., 20 cfu/mL) (D.lgs 31/2001) and its revision (DM 14/07/2017) [30,31].

Analysis of *P. aeruginosa* was performed using the standard membrane filter technique with culture on *Pseudomonas* selective agar (PSA, Biolife, Milan, Italy), according to UNI EN ISO 16266:2008 [32]. A volume of 100 mL was filtered for each sample using a cellulose nitrate membrane filter with a 0.45-µm pore size (Sartorius Stedim Biotech, Göttingen, Germany). Typical colonies of *P. aeruginosa* grown on selective media appeared as green colonies that were fluorescent under a Wood lamp (ultraviolet [UV] light at 280 nm). *P. aeruginosa* was also isolated and subjected to biochemical identification. Definitive identification was conducted using a BBL Crystal Enteric/Non-Fermenter ID Kit (Becton Dickinson Systems, Cockeysville, MD, USA), according to the manufacturer’s instructions, in addition to indole and oxidase reactions.

The results are expressed as cfu/100 mL, according to Italian drinking water regulation [30,31].

*Legionella* was isolated by a culture method according to ISO 11731:2017 [33]. For the enumeration of *Legionella* spp., different aliquots of the samples (untreated, filtered, and treated by heat and acid) were directly plated onto *Legionella* selective medium (*Legionella* glycine-vancomycin-polymyxin-cycloheximide [GVPC] selective medium, ThermoFisher Scientific, Oxoid, Ltd., Basingstoke, UK). All plates were incubated aerobically at 35.5 °C and 2.5% CO_2_ for up to 10 days. Colonies with the typical morphology of *Legionella* were enumerated and sub-cultured on buffered charcoal yeast extract (BCYE) agar, with and without cysteine. The isolates that grew on BCYE but failed to grow on the cysteine-free medium were verified serologically by an agglutination test (*Legionella* latex test kit, ThermoFisher Scientific, Oxoid, Ltd. Basingstoke, UK and polyclonal latex reagents, Biolife, Milan, Italy).

The results are expressed as cfu/L, according to the Italian Guidelines 2015 and Regional Guidelines publications [34,35]. The samples were considered negative when results were under the detection limit of the technique (<50 or 100 cfu/L).

### 2.4. Statistical Analysis

The data regarding the level of contamination for HPCs at 36 °C, *P. aeruginosa* and *Legionella* were converted into Log_10_ cfu/mL (Log cfu/mL), Log cfu/100 mL, and Log cfu/L, according to the directive limits. Statistical analyses were performed using SPSS software for Windows version 23 (IBM SPSS, Inc., Chicago, IL, USA). The Mann-Whitney test for nonparametric data was used to compare the parameters analyzed in the study. The data were considered significant for p values (*p*) ≤0.05.

## 3. Results

The data obtained showed that there were significant differences in terms of the level of contamination for all parameters tested between the two types of DUWLs (Type A vs. Type B). In Table 2, we show the data for the contamination found, expressed in terms of the percentage of positive samples (%), median, interquartile range (IQR), and range (minimal and maximum) of mean contamination for each parameter.

Regarding HPCs at 36 °C, samples that exceeded the bacterial contaminant concentrations prescribed by the regulations (500 cfu/mL according to CDC suggestions [26] and 20 cfu/mL according to D.lgs 31/2001 [30,31]) were considered positive. The rates of contamination in Type A DUWLs were 38.1% (32/84 samples) using the CDC criteria and 69.1% (58/84 samples) using the Italian D.lgs 31/2001 criteria. For Type B DUWLs, the rate of contamination with respect to the limits prescribed by the CDC was zero. In contrast, with respect to the Italian regulation criteria, the contamination rate was 56.3% (9/16 samples).

**Figure 1:**
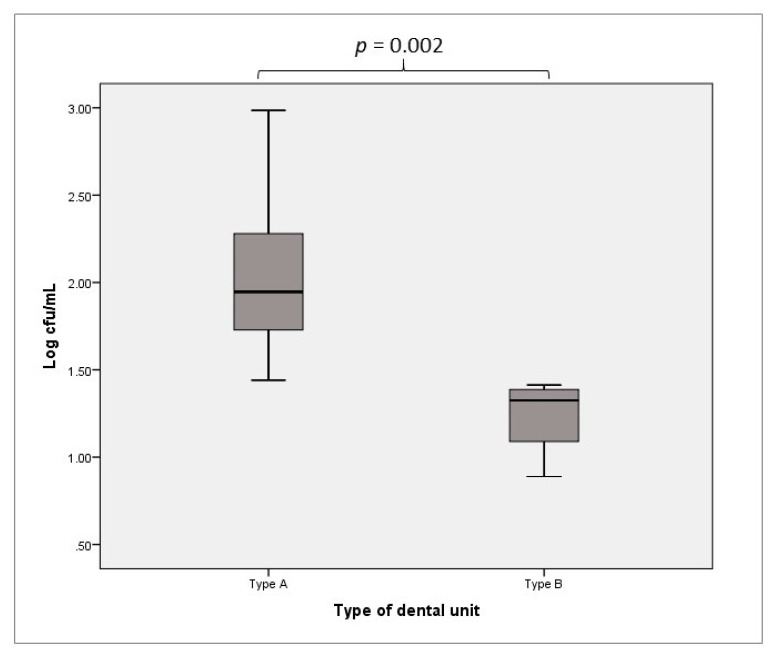
Heterotrophic plate counts (HPCs) at 36 °C: contamination of Type A vs. Type B DUWLs.

The bacterial contaminant concentrations of the samples from Type A DUWLs ranged from 1.44 to 2.99 Log cfu/mL, with median and IQR values of 1.95 Log cfu/mL and 0.63 Log cfu/mL, respectively. For Type B DUWLs, the range of mean concentrations was 0.89 to 1.41 Log cfu/mL, with a median of 1.33 Log cfu/mL and an IQR of 0.41 Log cfu/mL. The differences found were statistically significant (*p* = 0.002, Figure 1).

**Figure 2:**
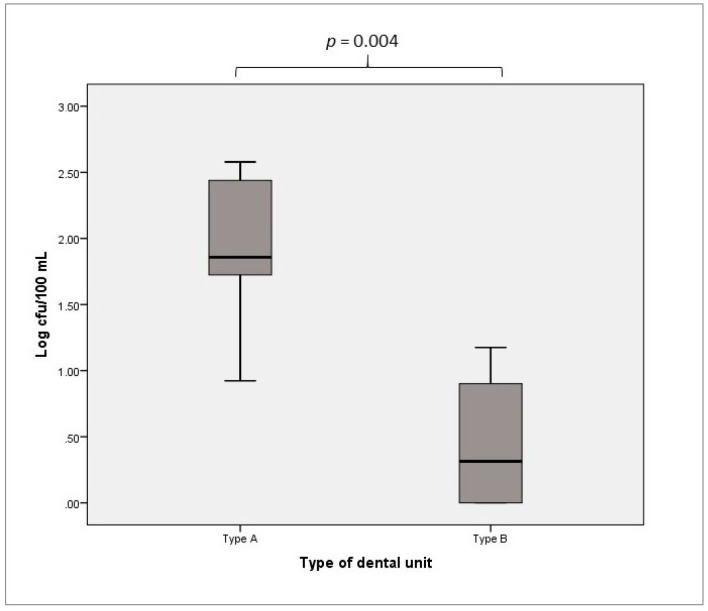
*P. aeruginosa* contamination of Type A vs. Type B DUWLs.

In accordance with the standards prescribed for drinking water, *P. aeruginosa* must be absent (0 cfu/100 mL) from DUWLs [30,31,36]. In line with previous results, we found a difference in terms of positive samples between Type A and Type B DUWLs. In particular, 65/84 (77.4%) Type A DUWL samples and 4/16 (25.0%) Type B DUWL samples showed positive results. The HPCs at 36 °C of the samples from Type A DUWLs ranged from 0.92 to 2.58 Log cfu/100 mL, with a median of 1.86 Log cfu/100 mL and an IQR of 0.73 Log cfu/100 mL. In contrast, the Type B DUWLs showed HPCs at 36 °C in the range of 0.00 to 1.17 Log cfu/100 mL, with a median of 0.31 Log cfu/100 mL and an IQR of 1.04 Log cfu/100 mL. The differences between the two types of DUWLs were statistically significant (*p* = 0.004, Figure 2).

Regarding contamination by *Legionella* spp., we observed the same trend as that for other parameters with respect to the percentage of positive samples and the level of contamination above the limit prescribed by the Italian Guidelines [33].

**Figure 3:**
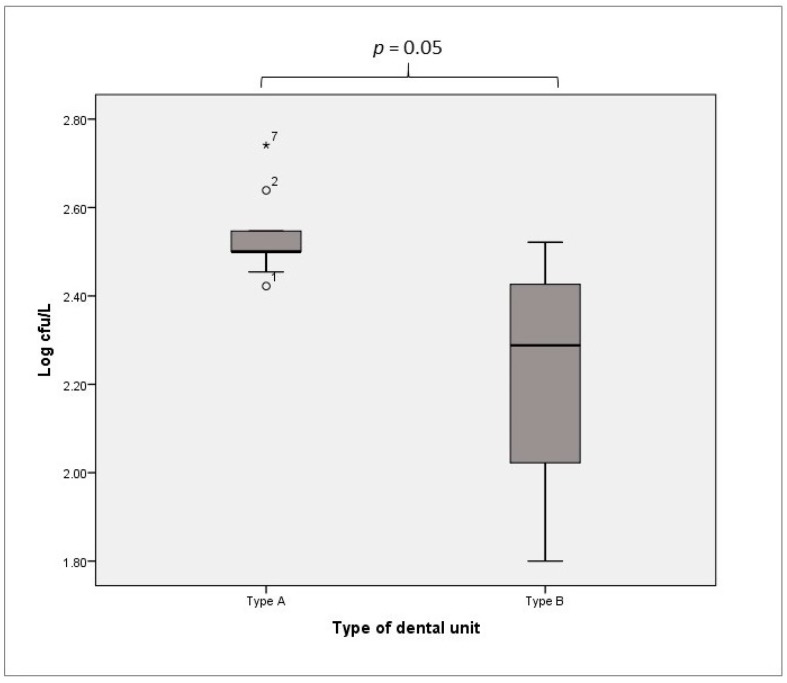
*Legionella* spp. contamination of Type A vs. Type B DUWLs.

*Legionella* was detected in 77/84 samples (91.7%) from Type A DUWLs and in 6/16 samples (37.5%) from Type B DUWLs. The samples from Type A DUWLs were positive, with values ranging from 2.42 to 2.74 Log cfu/L and with median and IQR values of 2.50 Log cfu/L and 0.08 Log cfu/L, respectively. The samples from Type B DUWLs were positive, with values ranging from 1.80 to 2.52 Log cfu/L and with median and IQR values of 2.29 Log cfu/L and 0.56 Log cfu/L, respectively. There was a significant difference in the detected levels of *Legionella* between Type A and Type B DUWLs (*p* = 0.05, Figure 3).

Isolates were subjected to phenotypical typing that permitted the identification of *Legionella pneumophila* serogroup 1 (SG1) and *Legionella* species in both types of DUWLs. The presence of *L. pneumophila* SG4 and SG8 was only detected in Type A DUWLs.

## 4. Discussion

The dental unit consists of a complex water-pipeline network connected to multiplex equipment, which represents an environment at high risk of microorganism transmission, especially through the contamination of water and bioaerosol production by dental instruments, which are placed extremely close to patients and medical staff during dental treatments.

The presence of microorganisms is linked to water supply characteristics and the presence of patients, who can transmit pathogenic or nonpathogenic organisms through their airflow. Although no epidemiologic evidence indicates a public health problem, the presence of substantial numbers of pathogens in DUWLs is a matter of concern [26].

The main problems related to dental unit contamination are linked to poor knowledge of the operators (medical staff and manufacturers) regarding water quality and the role of biofilm formation in DUWLs. Often, relevant information and adequate training on the proper use and maintenance of DUWL systems are missed.

Many guidelines and protocols have been established regarding procedures for DUWL maintenance and disinfection. Examples of such approaches include the flushing of waterlines for two to three minutes at the beginning of each day and for 20 to 30 seconds between each patient as well as the use of anti-retraction valves to prevent oral fluids from being drawn into dental waterlines and the adoption of sterile instrumentation. However, these guidelines and protocols lack standardization and provide only suggestions for combination approaches to ensure high-quality dental units [26,36,37].

In our study, the adoption of a water safety plan for the DUWL environment represents the first step toward understanding and controlling the risk of microbial contamination.

The results obtained indicate that the water supply can influence the presence of some species that are predominantly environmental organisms and Gram-negative nonfermenting bacteria (including *P. aeruginosa* and *Legionella* spp.), which are not only potential pathogens for humans but can contribute to biofilm formation within DUWLs, which increases the risk of infection.

Our investigation focused on routine monitoring of bacteria both as indicators of water quality and as human pathogens, which confirms, as suggested by previous studies, that the presence of microbial contamination in DUWLs represents a biological risk during DUWL treatment [37,38,39].

The novelty of the results is linked to the comparison of the microbial contaminants in two types of DUWLs: Type A, provided by bottle tanks close to dental chairs, and Type B, which is directly connected to municipal water networks.

Type A was found to be heavily contaminated relative to Type B for all parameters tested. In particular, with respect to HPCs at 36 °C, we found that Type A DUWLs always exceeded the reference values of both directives. In contrast, Type B DUWLs showed a low percentage of positive samples, with levels of contamination that were compliant with the CDC reference value (<500 cfu/mL) yet higher than the Italian reference value (<20 cfu/mL). This finding suggests that the use of specific directives can change the evaluation of water quality, with an underestimation of risk.

It is well documented that the monitoring of water quality is traditionally based on the determination of indicators that are not in and of themselves the cause of infections or diseases (e.g., the HPCs at 36 °C), but are organisms that should “predict” the potential risk related to the presence of pathogens [40]. This is caused because the relationships between the organisms’ concentrations and those of pathogens are never constant and because indicators are not predictive of infections [41]. Despite these considerations, our data indicating the presence of high levels of heterotrophic bacteria in Type A DUWLs suggest that the detection of HPCs at 36 °C represents a useful indicator of increased microbial growth, increased biofilm activity, extended retention times, water stagnation, and breakdown of the integrity of the system. This type of detection is often missed during routine environmental control and within critical water circuits such as dental units, which are characterized by a complex system design, low flow rates, and plastic materials [27,37,41].

These findings may be supported by the higher levels of the pathogens *P. aeruginosa* and *Legionella* in Type A than in Type B DUWLs. In agreement with other studies [38,42], we found that the presence of microflora (e.g., HPCs at 36 °C and Pseudomonadaceae) can interfere with *Legionella* growth and give false negative results. This evidence suggests the important role of monitoring for these microorganisms during environmental surveillance of *Legionella* in the dental unit.

*P. aeruginosa* is an important indicator of biofilm growth in DUWLs, and, according to the Italian directive for drinking water, this organism must be absent [30,31,36]. It is widely documented that this bacterium is a normal resident in the oral cavity but is able to cause dental infections in immunocompromised patients [4]. However, the evidence linking exposure to *P. aeruginosa* contaminated DUWLs during dental treatment and subsequent infection is limited and is based on the results from a single observational study [10]. Our results highlight the role of the routine introduction of *P. aeruginosa* into the DUWL water safety plan.

The presence of *P. aeruginosa* inside the water circuits of dental units is, in the majority of cases, connected to the phenomenon of retro-contamination. In this case, the bacteria move inside the tubes, and the small size of the tubes permits water stagnation and biofilm development. The competitive advantage of *P. aeruginosa* and *Legionella* spp. in the colonization of water lines is due to the capacity of *P. aeruginosa* to inhibit the growth of other bacteria by producing bacteriocins [43,44].

Directly connected with biofilm growth is the presence of *Legionella* spp. in dental water. These species are ubiquitously present in aquatic environments, including potable water supplies. DUWLs are a potential source of exposure to *Legionella* spp., especially from handpieces aerosols [45]. Regarding *Legionella* detection in dental circuits, after some documented cases associated with dental practices were found to occur in patients [46], the new version of the Italian Guidelines for the prevention and control of legionellosis included the dental unit environment for the first time. The guideline suggestions, supported by epidemiological data, introduce the obligation to perform a risk assessment as well as monitoring for the presence of *Legionella*, which is required annually. In the absence of specific limits for dental units, the guidelines suggest that *Legionella* levels be compliant with the reference values prescribed for hospitals [34,47].

The significant differences obtained from the analysis of the two types of DUWLs (Type A vs. Type B) is concerning since these two pathogens are likely associated with the type of water used by the dental unit (e.g., supplanted only by a bottle tank), the maintenance procedure applied, the absence of anti-retraction valves, and the type of bottle tank provided. The use of a closed circuit, such as an independent tank, which is found in Type A systems, may increase the risk of bacterial retro-contamination and the possibility that disinfection procedures will not be efficient.

A careful analysis of this type of dental unit permitted us to explain the results obtained. The medical staff mainly used water bottle tanks filled with distilled or sterilized water. Bottle tanks containing 1 or 2 L of water are not completely emptied after dental procedures, and the water is often not removed and drained. Water that remains in the tank overnight or during the weekend becomes a reservoir for bacterial growth. Furthermore, the possibility of switching between two different types of water flow (municipal water and sterile water) improves the risk of circuit contamination, but a mixed water supply is often not recorded in water safety plans.

We observed that bottle tanks are often composed of polyethylene (PE) or PTFE materials, to which microorganisms easily adhere to form biofilms. These materials are not capable of autoclaving or able to be treated with a high-activity disinfectant (e.g., peracetic acid) and exhibit visible damage such as rips and tears, which induce the formation of a bacterial niche where the disinfection procedure fails. Our analysis performed by swabbing a bottle tank (data not shown) confirms this hypothesis and suggests the role of a good disinfectant procedure, both in terms of the choice of disinfectant (liquid form is better) and the consideration of contact time to produce better results, which requires a minimum of 15 minutes [27,37].

The noncompliant results found in this study led to the discontinuation of dental unit activity, shock disinfection treatment, and resampling. However, these extraordinary procedures yielded unsatisfactory results over the long term, with a rapid recolonization of water lines.

In contrast, Type B DUWLs are supplied only by municipal tap water, which is subjected to routine monitoring, with the choice to remove the bottle tank from the water circuit. The dental chair provides a prefilter device, and a complex of anti-retraction valves is incorporated into all handpieces, ultrasonic scalers, and water lines to prevent backflow and to help restrain the contamination [27,37]. The lower percentage of positive samples and the lower level of contamination for all parameters tested also indicate that a different type of approach is used to clean the water circuit, which is easily flushed at the beginning of the working day and between each patient due to the possibility of using a greater quantity of water coming from the supply network. Furthermore, the presence of a special container that dispenses a liquid disinfectant (in a continuous fashion, at the end of each working day or after surgery procedures) favors cleaning operations, which ensures the correct contact time for the disinfectant throughout the water network. The disinfectant residue is discarded by the rewashing procedures with sterile or distilled water, which reduces the exposure of patients or medical staff. The half-yearly monitoring of tap water quality ensures the safety of the water supply.

The constant application of this protocol permits the maintenance of a hygienic and safe environment for dental treatments.

## 5. Conclusions

In our experience, the true contamination of the DUWL environment is often difficult to evaluate.

Some limitations for correctly estimating risk include the presence of a complex water pipeline, the difficulty of finding a specific disinfection procedure in terms of the disinfectant dosage and contact time, the possibility of carrying out the disinfection procedure during the working day (e.g., very short time between patients, poor staff training), and the presence of microflora that can interfere with the growth of some bacteria (e.g., *P. aeruginosa* vs. *Legionella*).

Our results from the evaluation of the microbial contamination of DUWLs direct attention to an “environment” in which the biological risk is often underestimated suggests the need for an integrative approach among manufacturers, medical staff, and public health stakeholders involved in institutional control. There is a real need for each dental unit to have a risk assessment plan containing comprehensive operational manuals in which all maintenance procedures, cleaning and inspection phases, and control of physical (disinfectants, temperature, flushing, etc.) and biological parameters (water quality) are periodically carried out and recorded.

The limitation of our study is linked to the set of observations performed only at the beginning of the working day. The possibility of testing the water quality at different time points (e.g., during medical activities, at the end of the working day, and after a long period of inactivity) may support our observations and permit the implementation of disinfection procedures on a new schedule.

Despite the low incidence of disease caused by exposure to dental unit water or air and the short-term benefits of disinfection approaches, disinfection appears to be the main strategy to reduce the risk of infection. Our data show that the detection of dental unit microflora can change in relation to the type of water and, especially, the procedures carried out, which indicates that water quality assessment is a valid approach to evaluate if a disinfection procedure is compliant with drinking water directives.

## Figures and Tables

**Table 1:** DUWL characteristics and maintenance protocols adopted.

Duwl Characteristics	Type A	Type B
Material	plastic-steel	plastic-steel
Water supply	storage tank/municipal water (button to switch the modality)	only municipal water supply
Anti-retraction valves	not present	present (changed monthly)
Storage capacity	1 or 2 L	not present—external container for disinfection practice
Flushing	twice a day (every day at the beginning and at the end of the working day and after each patient)	twice a day (every day at the beginning and at the end of the working day and after each patient)
Disinfection product/ procedure in use	treatment with chemical agents based on effervescent tablets *	treatment with hydrogen peroxide/silver salts (3% *v*/*v*) approved and patented by the manufacturer
Timing of disinfection	weekly (at the end of each working day) after a period of inactivity (weekends and holidays)	weekly (at the end of each working day) and after a period of inactivity (weekends and holidays)
Type of disinfection	sanitization is manually performed (1 or 2 tablets/1L or 2 L tank)	the sanitization operation is controlled by an automatized pump system (continuous treatment)
Problem with disinfection	possible retrograde contamination (no water pressure)	use of continuous treatment can show modest effectiveness and select for resistant microorganisms
Risk assessment for water control	every 6 months and/or following noncompliant results	every 6 months and/or following noncompliant results

* effervescent tablets formulation: sodium percarbonate (6.96%), dimethyl benzyl ammonium chloride (0.85%), dimethyl ethylbenzyl ammonium chloride (0.85%), silver nitrate (0.14%), and other ingredients (91.2%).

**Table 2:** Concentrations of microbiological contaminants in DUWL samples: Type A vs. Type B.

Microbiological Parameters	Statistical Parameters	Type A	Type B	Type A vs. Type B: *p*-value (One-Sided)
**HPCs at 36 °C**	% of positive samples (CDC 2003)	38.1%	0	0.002
	% of positive samples (D.lgs 31/2001)	69.1%	56.3%	
	Median, IQR	1.95, 0.63 Log cfu/mL	1.33, 0.41 Log cfu/mL	
	Range of the mean concentration (min-max)	1.44–2.99 Log cfu/mL	0.89–1.41 Log cfu/mL	
	Reference value: 500 cfu/mL (2.69 Log cfu/mL) Source of the reference value: CDC 2003 [26] Reference value: 20 cfu/mL (1.3 Log cfu/mL) Source of the reference value: Italian D.lgs 31/2001 [30,31]			
***P. aeruginosa***	% of positive samples	77.4%	25.0%	0.004
	Median, IQR	1.86, 0.73 Log cfu/100 mL	0.31, 1.04 Log cfu/100 mL	
	Range of the mean concentration (min-max)	0.92–2.58 Log cfu/100 mL	0.00–1.17 Log cfu/100 mL	
	Reference value: 0 cfu/100 Ml Source of the reference value: Italian D.lgs 31/2001 [30,31]			
***Legionella* spp.**	% of positive samples	91.7%	37.5%	0.05
	Median, IQR	2.50, 0.08 Log cfu/L	2.29, 0.56 Log cfu/L	
	Range of the mean concentration (min-max)	2.42–2.74 Log cfu/L	1.80–2.52 Log cfu/L	
	Reference value: 100 cfu/L (2 Log cfu/L) Source of the reference values: Italian Guidelines 2015 [34] Emilia Romagna Guidelines, 2017 [35].			

## References

[1] Blake G.C. (1963). The incidence and control of bacterial infection in dental spray reservoirs. Br. Dent. J..

[2] Rowland B.M. (2003). Bacterial contamination of dental Unit waterlines: What is your dentist spraying into your mouth?. Clin. Microbiol. Newsl..

[3] Barbot V., Robert A., Rodier M.H., Imbert C. (2012). Update on infectious risks associated with dental unit waterlines. FEMS Immunol. Med. Microbiol..

[4] Tuttlebee C.M., O’Donnell M.J., Keane C.T., Russell R.J., Sullivan D.J., Falkiner F., Coleman D.C. (2002). Effective control of dental chair unit waterline biofilm and marked reduction of bacterial contamination of output water using two peroxide-based disinfectants. J. Hosp. Infect..

[5] Ji X.Y., Fei C.N., Zhang Y., Zhang W., Liu J., Dong J. (2016). Evaluation of bacterial contamination of dental unit waterlines and use of a newly designed measurement device to assess retraction of a dental chair unit. Int. Dent. J..

[6] Putnins E.E., Di Giovanni D., Bhullar A.S. (2001). Dental unit waterline contamination and its possible implications during periodontal surgery. J. Periodontol..

[7] Meiller T.F., Kelley J.I., Baqui A.A., DePaola L.G. (2000). Disinfection of dental unit waterlines with an oral antiseptic. J. Clin. Dent..

[8] Pankhurst C.L., Johnson N.W., Woods R.G. (1998). Microbial contamination of dental unit waterlines: The scientific argument. Int. Dent. J..

[9] Szymanska J. (2003). Biofilm and dental unit waterlines. Ann. Agric. Environ. Med..

[10] Pankhurst C.L., Coulter W.A. (2007). Do contaminated dental unit waterlines pose a risk of infection?. J. Dent..

[11] Fotedar S., Ganju S. (2015). Microbial contamination of dental unit water lines in H.P. Government Dental College, Shimla. Saudi J. Dent. Res..

[12] Uzel A., Cogulu D., Oncag O. (2008). Microbiological evaluation and antibiotic susceptibility of dental unit water systems in general dental practice. Int. J. Dent. Hyg..

[13] Ampornaramveth R.S., Akeatichod N., Lertnukkhid J., Songsang N. (2018). Application of D-Amino Acids as Biofilm Dispersing Agent in Dental Unit Waterlines. Int. J. Dent..

[14] Pederson E.D., Stone M.E., Ragain J.C., Simecek J.W. (2002). Waterline biofilm and the dental treatment facility: A review. Gen. Dent..

[15] Abdouchakour F., Dupont C., Grau D., Aujoulat F., Mournetas P., Marchandin H., Parer S., Gibert P., Valcarcel J., Jumas-Bilak E. (2015). *Pseudomonas aeruginosa* and *Achromobacter* sp. clonal selection leads to successive waves of contamination of water in dental care units. Appl. Environ. Microbiol..

[16] Szymańska J., Sitkowska J., Dutkiewicz J. (2008). Microbial contamination of dental unit waterlines. Ann. Agric. Environ. Med..

[17] O’Donnell M.J., Boyle M.A., Russell R.J., Coleman D.C. (2011). Management of dental unit waterline biofilms in the 21st century. Future Microbiol..

[18] Garg S.K., Mittal S., Kaur P. (2012). Dental unit waterline management: Historical perspectives and current trends. J. Investig. Clin. Dent..

[19] Coan L.L., Hughes E.A., Hudson J.C., Palenik C.J. (2007). Sampling water from chemically cleaned dental units with detachable power scalers. Am. Dent. Hyg. Assoc..

[20] Lin S.M., Svoboda K.K.H., Giletto A., Seibert J., Puttaiah R. (2011). Effects of hydrogen peroxide on dental unit biofilms and treatment water contamination. Eur. J. Dent..

[21] Ditommaso S., Giacomuzzi M., Ricciardi E., Garbuio R., Zotti C.M. (2018). The role of chemical products at low doses in preventing the proliferation of bacteria in dental unit waterlines: The ICX® experience. J. Water Health.

[22] Nante N., Ceriale E., Messina G., Lenzi D., Manzi P. (2017). Effectiveness of ATP bioluminescence to assess hospital cleaning: A review. J. Prev. Med. Hyg..

[23] World Health Organization (2014). Water Safety in Distribution Systems. http://www.who.int/water_sanitation_health/publications/Water_safety_distribution_systems_2014v1.pdf.

[24] USEPA (United States Environmental Protection Agency) (2006). Distribution System Indicators of Drinking Water Quality.

[25] National Research Council (2006). Drinking Water Distribution Systems: Assessing and Reducing Risks.

[26] William G., Kohn A.S., Collins M.P.H., Jennifer L., Cleveland D.D.S., Jennifer A., Harte D.D.S., Kathy J., Eklund M.H.P., Dolores M. (2003). Centre for Disease Prevention and Control (CDC). Guidelines for Infection Control in Dental Health-Care Settings—2003. http://www.cdc.gov/mmwr/PDF/rr/rr5217.pdf.

[27] Health Technical Memorandum: Decontamination in Primary Care Dental Practices (HTM 01-05, 2013 Version). https://www.gov.uk/government/publications/decontamination-in-primary-care-dental-practices.

[28] Hwang C., Ling F., Andersen G.L., LeChevallier M.W., Liu W.T. (2012). Microbial community dynamics of an urban drinking water distribution system subjected to phases of chloramination and chlorination treatments. Appl. Environ. Microbiol..

[29] UNI EN ISO 6222:2001 Water Quality—Enumeration of Culturable Micro-Organisms—Colony Count by Inoculation in a Nutrient Agar Culture Medium. http://store.uni.com/catalogo/index.php/uni-en-iso-6222-2001.html.

[30] Decreto Legislativo 2 Febbraio 2001, n. 31, Attuazione Della Direttiva 98/83/CE Relativa Alla Qualità Delle Acque Destinate al Consumo Umano (G.U. n. 52 del 3 marzo 2001-s.o.n. 41). http://www.gazzettaufficiale.it/eli/id/2001/03/03/001G0074/sg.

[31] (2017). Ministerial Decree 14.06.2017. Implementation of Directive (EU) 2015/1787 Amending Annexes II and III of Directive 98/83/EC on the Quality of Water Intended for Human Consumption. Amendments to Annexes II and III of Legislative Decree 2 February 2001, n. 31. (17A05618) (GU General Series n.192 of 18-08-2017). https://www.google.com/url?sa=t&rct=j&q=&esrc=s&source=web&cd=3&ved=2ahUKEwis_Nm09ZTfAhUKw4sKHRPbAFAQFjACegQIAhAC&url=http%3A%2F%2Fwww.ceirsa.org%2Ffd.php%3Fpath%3D201710%2FDM_14_06_2017Acque_destinate_al_consumo_umano.pdf&usg=AOvVaw3eguX4JKnVJArmnYdE1oVD.

[32] UNI EN ISO 16266:2008—Water Quality—Detection and Enumeration of *Pseudomonas aeruginosa*—Method by Membrane Filtration. http://store.uni.com/catalogo/index.php/uni-en-iso-16266-2008.html?josso_back_to=http://store.uni.com/josso-security-check.php&josso_cmd=login_optional&josso_partnerapp_host=store.uni.com.

[33] ISO 11731:2017 Water Quality—Enumeration of Legionella. https://www.iso.org/standard/61782.html.

[34] Italian Health Ministry (2015). Guidelines for Prevention and Control of Legionellosis. Approvate in Conferenza Stato-Regioni Seduta Del 7 Maggio 2015. http://www.salute.gov.it/imgs/C_17_pubblicazioni_2362_allegato.pdf.

[35] Emilia-Romagna Region (2017). Regional Guidelines for Surveillance and Control of Legionellosis. Delibera Della Giunta Regionale 12 Giugno 2017, N. 828. http://salute.regione.emilia-romagna.it/documentazione/leggi/regionali/dgr-2127-2016/dgr-n-828-2017-linee-guida-regionali-per-la-sorveglianza-e-il-controllo-della-legionellosi/view.

[36] World Health Organization (2011). Guidelines for Drinking-Water Quality—4th ed. http://apps.who.int/iris/bitstream/10665/44584/1/9789241548151_eng.pdf.

[37] ADA (2004). American Dental Association: Statement on Dental Unit Waterline. http://www.ada.org/.

[38] Pasquarella C., Veronesi L., Castiglia P., Liguori G., Montagna M.T., Napoli C., Rizzetto R., Torre I., Masia M.D., Di onofrio V. (2010). Siti workinggroup “Hygiene in Dentistry”. Italian multicentre study on microbial environmental contamination in dental clinics: A pilot study. Sci. Total Environ..

[39] Sedlata Juraskova E., Sedlackova H., Janska J., Holy O., Lalova I., Matouskova I. (2017). *Legionella* spp. in dental unit waterlines. Bratisl. Lek. Listy.

[40] Duda S., Baron J.L., Wagener M.M., Vidic R.D., Stout J.E. (2015). Lack of correlation between *Legionella* colonization and microbial population quantification using heterotrophic plate count and adenosine triphosphate bioluminescence measurement. Environ. Monit. Assess..

[41] Bristela M., Skolka A., Schmid-Schwap M., Piehslinger E., Indra A., Wewalka G., Stauffer F. (2012). Testing for aerobic heterotrophic bacteria allows no prediction of contamination with potentially pathogenic bacteria in the output water of dental chair units. GMS Krankenhaushygiene Interdisziplinär.

[42] Ditommaso S., Giacomuzzi M., Ricciardi E., Zotti C.M. (2016). Cultural and Molecular Evidence of *Legionella* spp. Colonization in Dental Unit Waterlines: Which Is the Best Method for Risk Assessment?. Int. J. Environ. Res. Public Health.

[43] Wilson R., Dowling R. (1998). Lung infections. 3. Pseudomonas aeruginosa and other related species. Thorax.

[44] Kimura S., Tateda K., Ishii Y., Horikawa M., Miyairi S., Gotoh N., Ishiguro M., Yamaguchi K. (2009). *Pseudomonas aeruginosa* Las quorum sensing autoinducer suppresses growth and biofilm production in *Legionella species*. Microbiology.

[45] Coleman D.C., O’Donnell M.J., Shore A.C., Russell R.J. (2009). Biofilm problems in dental unit water systems and its practical control. J. Appl. Microbiol..

[46] Ricci M.L., Fontana S., Pinci F., Fiumana E., Pedna M.F., Farolfi P., Bucci Sabattini M.A., Scaturro M. (2012). Pneumonia associated with a dental unit waterline. Lancet.

[47] Rota M.C., Caporali M.G., Bella A., Ricci M.L., Napoli C. (2013). Legionnaires’ disease in Italy: Results of the epidemiological surveillance from 2000 to 2011. Eurosurveillance.

